# On the Use of Oxidation Induction Time as a Kinetic Parameter for Condition Monitoring and Lifetime Evaluation under Ionizing Radiation Environments

**DOI:** 10.3390/polym14122357

**Published:** 2022-06-10

**Authors:** Eduard-Marius Lungulescu, Radu Setnescu, Sorin Ilie, Mauro Taborelli

**Affiliations:** 1 National Institute for Research and Development in Electrical Engineering ICPE-CA, 313, Splaiul Unirii, 030138 Bucharest, Romania; radu.setnescu@icpe-ca.ro; 2 Department of Advanced Technologies, Faculty of Sciences and Arts, Valahia University of Târgoviște, 13 Aleea Sinaia, 130004 Targoviste, Romania; 3 TS-MME/CCS in European Organization for Nuclear Research (CERN), 1211 Geneva, Switzerland; sorins712@gmail.com; 4 European Organization for Nuclear Research, 1211 Geneva, Switzerland; mauro.taborelli@cern.ch

**Keywords:** polymeric insulators, gamma irradiation, OIT, antioxidants, DSC, kinetic model, lifetime assessment

## Abstract

The durability of polymeric materials is closely linked to their degradation under specific operating conditions when different stressors—general or specific, such as high temperature, sunlight or ionizing radiation, solvents, or mechanical stresses—act simultaneously, causing degradation. In the case of electrical cables, the durability of the electrically insulating materials used in their construction is an important parameter to ensure their operational security. In this work, we studied the degradation state of various types of electrical insulating materials from cables used in particle acceleration systems under European Organization for Nuclear Research (CERN) conditions (e.g., Super Proton Synchrotron, SPS) as a function of time and irradiation dose. A simple kinetic model was proposed based on the exponential decrease in the antioxidant amount in polymeric insulations. The onset oxidation time (OIT) values, used as an indicator of antioxidant concentration, were obtained from isothermal differential scanning calorimetry (DSC) and chemiluminescence (CL) measurements. Fourier transform infrared (FTIR) measurements were used to assess the degradation state and identify polymeric materials. The practical applicability of such a model in diagnosing degradation and in the subsequent evaluation of the remaining service life is of interest, as it can be adapted to a broad range of operating conditions and materials.

## 1. Introduction

Condition monitoring and the lifetime evaluation of different products that incorporate polymer materials are important research directions for applications in electrical engineering and related fields. Because of their organic structure, most polymer materials are quasi-unstable under environment-specific exploitation conditions, e.g., elevated temperatures, solar radiation, electric fields, ionizing radiations, mechanical stress, etc. Most real cases include the presence of atmospheric oxygen and combinations of the factors mentioned above in varying proportions, which can complicate predictions. Therefore, significant differences would be frequently observed between the laboratory and real cases because of differences between the simulated laboratory conditions and the real world, e.g., different temperature ranges or cycles, different dose rate values, or combined action of different stress factors during specific periods [1,2,3].

The lifetime of polymeric materials would not be so intensely studied if the condition of the polymeric materials did not decisively influence the functionality of the more complex aggregates that incorporate these materials, such as electrical cables, pipes carrying liquids under pressure, various structural elements, etc. Several polymers are frequently used in cable production due to their good dielectric properties and chemical inertness. Polyethylene, under different grades, is, by far, the most used material in the fabrication of the insulators of general-use cables and sometimes of external sheaths. Various other copolymers, such as ethylene-propylene copolymer (EPR), ethylene-vinyl acetate copolymer (EVA), and natural or synthetic rubbers, are used in different applications. Polyvinyl chloride (PVC) and halogen-containing polymers are becoming more restricted due to toxic products evolved during their thermal decomposition/burning. High-temperature polymers, such as fluorinated ethylene-propylene copolymer (FEP), polyimide film (Kapton), and polyimide resins, are used in special cable manufacture due to their excellent electrical and chemical resistance and good mechanical properties [4,5,6].

Among these polymers, the hydrocarbon-based ones (PE, EPR, EVA, and natural and synthetic rubbers) are relatively susceptible to degradation under environmental conditions; therefore, antioxidants and stabilizers are used to suppress the various degradation processes, mainly the oxidative processes, expanding their lifespan [7,8,9]. The presence of an antioxidant within a hydrocarbon polymer matrix can be easily detected by isothermal oxidative tests through thermal analysis methods (DSC, chemiluminescence) when a significantly longer induction period of oxidation is observed compared to the blank (free of antioxidants) polymer. Hence, the oxidation induction time (OIT) or its corresponding non-isothermal (temperature ramp) measurement, i.e., oxidation onset temperature (OOT), can be used for characterization of the oxidative stability and is understood to be related in a particular manner to the lifetime of that material [10,11,12,13].

There are several proposed experimental methods for diagnosing the degradation state of a certain polymer material. Some of these methods do not require specific sampling (e.g., determination of the indentation module, medium, and near infrared spectroscopies). In contrast, other methods require microsamples (e.g., determination of the modulus of elasticity profile, IR spectroscopy for laboratory testing, thermogravimetry analysis, density measurements, gel fraction determination, and solvent absorption) [14,15,16,17,18,19]. Many evaluations are based on the measurement of physicochemical properties (such as infrared spectra, melting/crystallization temperature [20], glass transition temperature or mechanical tests [21], mass loss during laboratory aging [22], or electrical tests) rather than on oxidation induction time (OIT) tests. At the beginning of aging, many physical parameters (including IR bands, mechanical and electrical properties, crystallinity, mass loss, etc.) do not change, or their variations are nonlinear, making it difficult to use them in long-term evaluations. The worsening of these parameters is undoubtedly related to the deterioration of the polymer, but this relation is frequently complicated because the dependence of the degradation parameter on aging time is, itself, often complicated.

Some of the most important methods for evaluating the lifetime of polymeric materials are based on Arrhenius-type models. These methods require accelerated aging tests at high temperatures, at which the speed of the oxidative processes is reasonably high for each temperature and stress time, and then the value of the degradation parameter is determined. Arbitrary setting of a limit criterion for the degradation parameter, for example, reaching 50% of the elongation at break, allows for the kinetic treatment of aging data [23,24]. Thus, by representing this time as a function of the inverse of the aging temperature, a straight line will be obtained, which can be extrapolated to lower temperatures specific to material exploitation and the value of the time at which the degradation parameter reaches its minimum value, allowing for the evaluation of the lifetime of the given material.

Standardized evaluation methods based mainly on the worsening of mechanical properties have been proposed for cables and insulating materials [19,25]. However, for some applications (as in the case of particle accelerators or nuclear power plants), the operating parameters vary on a large scale. The initial conditions may change during the exploitation, making assessment of the cable’s initial lifetime difficult. Besides the time and the human resource consumption required for classical mechanical tests, a complex problem in monitoring cable behavior arises from the relatively high amounts of cable material necessary for such periodic mechanical tests. Moreover, the amount of available material sample from a given aged item is frequently very low, and the space for whiteness materials is also drastically limited. Hence, monitoring and evaluating a cable’s residual lifetime in situ becomes essential in the development of safety strategies [26].

Outstanding pioneering work in lifetime estimation of materials for nuclear power plants was done by Clogh and Gillen, who proposed the use of accelerating factors for aging under multiple stress conditions (temperature and radiation) within the time–temperature–dose rate superposition methodology (see for example [27]), as well as by Schonbacher and Tavlet at CERN, who proposed initial qualification of different materials (based on mechanical tests) for use in radiation environments [28,29].

More elaborated kinetic models are based on kinetic constants related to the specific degradation mechanisms [13,30]. As such models include rate constants of the processes involved in degradation, they are very accurate in lifetime prediction. However, owing to the many approximations necessary to simplify the calculations, such as neglecting or considering simplified equations for stabilizer evaporation or for the temperature dependence of evaporation rate from a polymer matrix, the concordance with real aging cases is sometimes unsatisfactory. The influence of the polymer molecular structure and morphology on either the degradation mechanism or additive compatibility could be another cause of the inconsistency of the models relative to real cases. Other limiting factors in the development of both reliable and simple kinetic models include input data, such as the values of some kinetic constants, which, if material-dependent (determined previously in time-consuming laboratory experiments), result in a material-dependent model because of the specific values of the coefficients and constants [31].

Besides the abovementioned problems, methods based on extrapolation could present further disadvantages, such as: (i) the oxidation mechanism is not constant over a wide temperature range [24]; (ii) the results of extrapolations performed over physical transition points (such as melting of crystallinity), as well as accelerated aging experiments at temperatures in the range of these transitions, may be affected by significant errors [24], as the oxidation mechanism changes as crystalline domains are formed and grow with decreased temperature; (iii) the occurrence of limited oxygen diffusion processes, which can result in significant deviations from the Arrhenius-type dependence [25].

This paper is devoted to the analysis of experimental data of isothermal DSC and chemiluminescence (CL) collected from samples of various materials aged under different stress conditions, mainly in radiation fields and air. The aim is to show the applicability of some simple kinetic considerations and a degradation model for ethylene-based polymer materials. The proposed kinetic model can evaluate the maximum irradiation dose and the residual lifetime of polymer materials for a cable in use. Furthermore, this kinetic model can be applied to determine the lifetime of polymer materials exposed to other environmental factors, such as elevated temperatures, UV exposure, natural weathering, etc.

## 2. Materials and Methods

### 2.1. Materials

The polyethylene-based materials used in this study, namely low-density polyethylene (LDPE), linear low-density polyethylene (LLDPE), high-density polyethylene (HDPE), crosslinked polyethylene (XLPE), ethylene-propylene-diene copolymer (EPR), and ethylene vinyl acetate copolymer (EVA), were obtained from two main sources: laboratory-available polymer samples and samples taken from various types of commercially available electric cables (instrumentation and control, high voltage and medium voltage). Most of the studied cables were used in CERN’s particle acceleration systems (e.g., Super Proton Synchrotron (SPS) and Large Hadron Collider (LHC)). For the structure of the cables studied in this work, as well as for the nature of the incorporated materials, please see the Supplementary Material. The commercial names of the polymers used in cable fabrication, as well as their producers, cannot be disclosed for commercial reasons. However, all the cables and the polymers used in their construction were produced in Europe.

### 2.2. Irradiation

The materials discussed here were aged under different conditions, and their oxidation kinetics before and after exposure were compared. For example, the aging effect of radiation fields was studied on either material samples previously separated from the cables or on materials sampled from irradiated cables. Gamma ^137^Cs irradiation was applied at ICPE-CA (dose rate: 0.3 kGy/h in either air or nitrogen as inert atmosphere), whereas ^60^Co irradiation was applied at Ionisos (Dagneaux, France; dose rate: 1.5 kGy). Cable witnesses were exposed in pre-established positions along CERN SPS and LHC accelerators, and periodically, the irradiated witnesses were sampled for condition monitoring of the real cables installed in the respective area.

### 2.3. Characterization Methods

**Figure 1:**
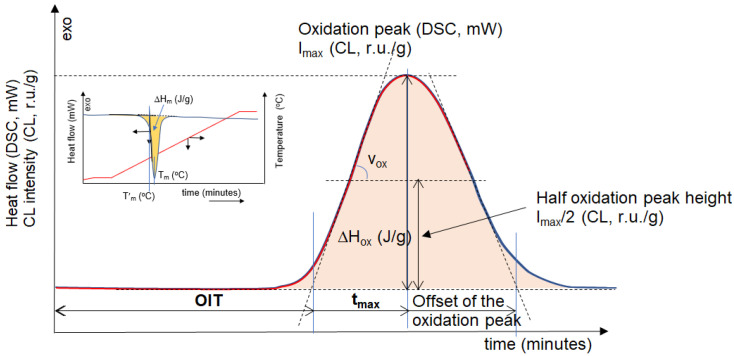
Typical isotherm curves of oxidation in DSC and CL measurements indicating the kinetic parameters that can be calculated from these curves: OIT, t_max_ (time to maximum of oxidation peak in either DSC and CL), and t_1/2_ (time to reach half of the oxidation peak) are the main parameters used in this work; other parameters include the oxidation rate (v_ox_ = the slope of the tangent at the inflexion point of the ascendent peak edge), offset of the oxidation peak, and the thermal effect of oxidation (ΔH_ox_). The inset shows a typical thermogram of the non-isothermal heating (10 °C/min) of a polyethylene sample in an inert (nitrogen) atmosphere in DSC; the melting endotherm can be observed; the parameters used for characterization of the melting process are the melting temperature (T_m_ or T′_m_) and the melting endotherm (ΔH_m_).

The oxidation induction time (OIT) was determined from DSC and chemiluminescence measurements under similar temperature conditions (isothermal mode). DSC measurements were performed with Setaram DSC 131 (Setaram, Caluire, France) at CERN and Setaram DSC 131 EVO (Setaram, Caluire, France) at CERN and INCDIE ICPE-CA for comparison studies. OIT from DSC was obtained according to [32]. Similar curves of *signal* vs. *temperature* were obtained with a Lumipol 3 (Polymer Institute of Slovak Academy of Sciences, Bratislava, Slovakia) chemiluminescence instrument with kinetic parameters of significance similar to those of DSC’s (Figure 1).

For DSC measurements, the samples (either as thin chops or slices with 0.2 mm thickness cut with a Jasco HS-1 vertical slicer (Jasco Corp., Tokyo, Japan)) were warmed from room temperature to the measurement temperature under nitrogen flow (50 mL/min) with a typical heating rate of 10 °C/min (see the heating diagram in Figure 1, inset). This procedure avoided sample degradation under warming and enabled the measurement of the typical transition temperatures (crystallinity melting).

Typical bands related to oxidation, namely carbonyl (1680–1730 cm^−1^), hydroxyl as alcohol and hydroperoxide groups (~3300–3500 cm^−1^), -C-O (~1068 and 1130 cm^−1^), and C=C (965–880 cm^−1^), were monitored [33,34,35]. Moreover, FTIR information was corroborated by DSC data for polymer identification fir each cable material. JASCO FTIR—4200 (Jasco Corp., Tokyo, Japan) or Bruker Vertex 70 FT-IR (Bruker, Marne la Vallee, France) spectrometers were used for sample recording (spectral range, 4000–400 cm^−1^; resolution, 4 cm^−1^). Due to opacity caused by the complex composition of the studied materials, in many cases, the infrared spectra were recorded with ATR (attenuated total reflectance) technique, using adequate ATR modules coupled to the mentioned spectrometers.

## 3. Results and Discussion

### 3.1. Kinetic Model: Methodology and Premises

Degradation of polymers is a complex process that involves changing their physical and chemical structure under the action of various stressors, either physical (e.g., heat, light, ionizing radiation, mechanical stress, ultrasound, solvents, etc.) or chemical (e.g., oxygen, ozone, metal ions, various chemical compounds, etc.) [36,37]. As a result, the useful properties of polymers can degrade [38,39].

**Scheme 1:**
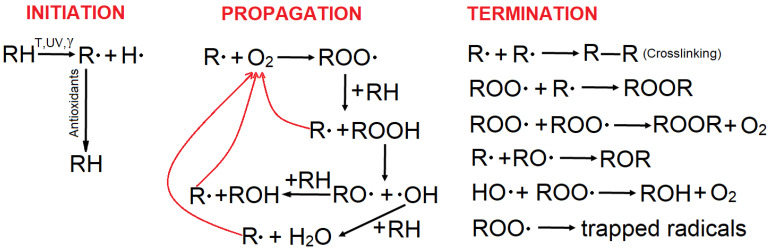
Oxidation degradation mechanism (adapted from [42,43]).

It is widely accepted that the most important and frequent form of degradation of polymeric materials is oxidation. The organic nature of polymers makes them susceptible to oxidative degradation under ambient conditions. The oxidative degradation processes of different polymers are governed by specific mechanisms determined by the chemical peculiarities of the main chain and the adjacent groups. [40,41]. In general, oxidative degradation of polymers occurs in their amorphous zone, and for many usual polymers, such as polyethylene, polypropylene, ethylene-based copolymers, and natural and synthetic rubbers, the degradation mechanism involves a chained process based on free radicals and hydroperoxide reactions as precursors of oxidation degradation products [42]. Note that the polymer examples mentioned above contain only carbon atoms within their main backbone (also called hydrocarbon polymers). Their oxidation mechanisms are similar and can be described by the so-called Bolland–Gee scheme [34] shown in Scheme 1.

In order to suppress the environmental degradation of the mentioned polymers and to extend their service life, stabilizing additives are used [44,45]. Antioxidants are largely used with many polymers because of their capacities to either annihilate free radicals (radical scavenger-type antioxidants) or decompose hydroperoxides in a way that is not harmful to the polymer. Hence, the chain-breaking antioxidants (e.g., phenols, amines, quinones, etc.) compete the reaction of free alkyl radicals (R^●^) with oxygen; other free radicals, such as peroxyl (ROO^●^) and alkoxyl (RO^●^), can also be annihilated.

As a consequence of antioxidant interference in the oxidation mechanism, an induction period of oxidative degradation is observed with stabilized polymers as compared to their antioxidant-free counterparts. This induction period is clearly observable in thermal analysis of isothermal measurements, such as DSC and CL, performed in oxidative atmospheres (e.g., air or oxygen). The duration of the induction period is proportional to the concentration of the antioxidant and decreases with increasing the temperature. When measured in an isothermal mode during thermal analysis, the oxidation induction period is called *oxidation induction time* (OIT).

**Figure 2:**
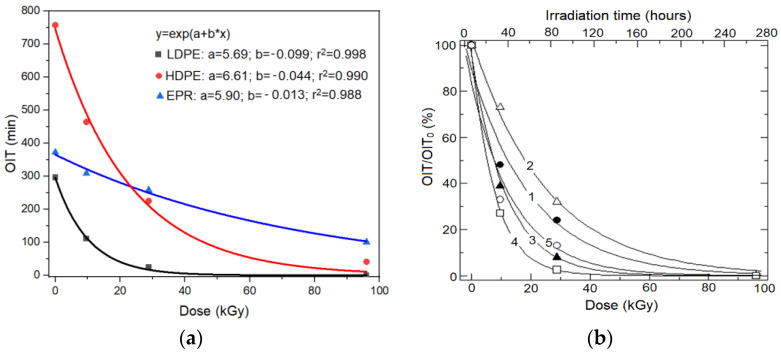
(**a**) Typical exponential decrease in OIT values with irradiation dose. OIT values were isothermally recorded at 190 °C (LDPE) and 200 °C (HDPE and EPR). (**b**) Decay of the normalized OIT values (OIT_D_/OIT_0_; D = the radiation dose) vs. γ-irradiation dose (^137^Cs, D_r_ = 0.4 kGy/h) for different LDPE insulation materials: 1—C1 * cable (irradiated as strap; Figure S1); 2—C4 cable (irradiated as bulk; Figure S2); 3—C3 cable (irradiated as strap; Figure S3); 4—C9 cable, white insulation (irradiated as bulk-encapsulated; Figure S4), 5—C9 cable, white insulation (irradiated as bulk-free ends, Figure S4); * laboratory codes.

Given the above considerations, the proposed methodology for the kinetic model is based on the following premises:

(a) The effect of the stress factors, irrespective of their nature, consists of molecular scissions, leading to free radicals. These species react rapidly with the surrounding oxygen in air and trigger oxidation chain reactions (Scheme 1), leading to material deterioration and implicitly shortening their lifetime. In the case of ethylene polymers, lifetime diminution is caused by the progressive consumption of antioxidants.
  The initiation of degradation (produced by breaking of labile chemical bonds of the polymer) occurs by the action of different stress factors (high temperatures, light, radiation, electric field, etc.). The formed free radicals are subsequently trapped by oxygen, and the degradation propagates as chain reactions of free radicals thus formed. On average, each free radical formed subsequently involves the reaction of five oxygen molecules [46]. The second component of propagation consists of reactions of hydroperoxides.
  Even in the absence of oxygen, stress factors can induce free radicals, producing polymer degradation by scission and eventual crosslinking. The subsequent air exposure of such material results in its oxidation due to the reactions of the trapped free radicals [47].
(b) Antioxidants (together with other additives) are chemically consumed in the reactions with the free radicals. Hence, a continuous depletion of the antioxidant content initially introduced in the polymer occurs during the service time of the material. During this time, no material deterioration occurs (or it is negligible), with the antioxidants acting as chemical barriers against the spreading of the oxidation process.
(c) The OIT values, which are determined at high temperatures specific to the test, correspond to the lifetime of the material under the conditions of exposure. The OIT value is directly proportional to the concentration of antioxidants in the polymeric material [2,35,45]. Therefore, OIT measurement can be used to “titrate” the antioxidant content of the sample. Thus, decreasing kinetics of OIT can be used to describe the kinetics of antioxidant depletion. Given the direct connection between OIT and material lifetime, decreasing OIT kinetics can be used to evaluate the lifetime at moderate temperatures specific to service conditions. As previously mentioned, the main problems for extrapolations are (i) the existence of a transition point (e.g., melting point of crystallinity), which can cause changes in the degradation mechanism; and (ii) the influence of temperature on the rates of different reactions. Hence, the degradation mechanism is expected to be different at higher temperatures as compared to moderate temperatures (due to the activation or deactivation of specific reactions).
(d) We hypothesize that under similar (quasi-constant) exposure conditions of a material, the resulting OIT reduction is proportional to the time spent in mild conditions specific to aging in real conditions. Thus, OIT can be used as a revealing agent of the degradation effect produced under mild conditions and not as an accelerated aging test. In this case, the lifetime of the material under real conditions is equal to the exposure time resulting in a zero OIT value.
  The above hypothesis was confirmed by experimental data (see Figure 2), enabling a “bypass” of the problem of extrapolation, with the OIT used as a “marker” of the remaining lifetime of the material.

Figure 2a shows that the experimental data present a progressive decrease in the OIT with an increase in the irradiation time for three different types of materials. The experimental points can be fitted well to an exponential decay. This behavior appears attractive for predictions because it corresponds to a first-order kinetics. Hence, the lifetime, which corresponds to the exposure time (under service conditions) until the OIT reaches a zero value (under the laboratory test conditions), can be easily estimated. This behavior has been experimentally observed for polymeric materials based on single ethylene monomers or their mixtures (copolymers), which follows a Bolland–Gee-type oxidation scheme (this scheme does not apply to polystyrene, which preponderantly decomposes, even under oxidative conditions at elevated temperatures).

A similar decrease in OIT with irradiation dose was observed for other insulation materials under controlled-dose irradiation conditions (Figure 2b), irradiated either as straps or as a bulk cable from where the samples for DSC measurements were subsequently taken.

In many practical cases of long-term exposures, the intensity of the factors inducing degradation can be considered quasi-constant (as an average), although their momentary cyclical variations may be significant; hence, the above kinetic considerations could be extended to such cases as well.

### 3.2. Kinetic Model for Evaluating the Lifetime of Polymeric Materials

If we consider the process of antioxidant depletion (1), where A is the antioxidant:

A → products (antioxidant degradation products)(1)

The rate of antioxidant consumption is described by the rate of OIT decrease and is given by Equation (2):

(2) $$-\frac{d\left[\mathrm{OIT}\right]}{\mathrm{dt}}=k\left[\mathrm{OIT}\right]$$

where [OIT] is the momentary value of OIT (which is proportional to the antioxidant concentration at a given time, *t*), and *k* is the rate constant.

Integration of Equation (2) leads to Equation (3):

(3) −ln[OIT]=k·t+C

where *C* is the integration constant, the chemical significance of which is obtained if *t* = 0 (initial reaction time); and *C* is the natural logarithm of the initial OIT value:

(4) $$C=-\ln{\left[\mathrm{OIT}\right]}_{0}$$

If *C* is substituted in Equation (3), the relation between the antioxidant concentration and the time (of aging) becomes:

(5) $$\left[\mathrm{OIT}\right]={\left[\mathrm{OIT}\right]}_{0}\cdot \;e^{-kt}$$

In the case of ionizing radiation aging, the parameter *t* can be replaced by the integrated dose, *D*:

(6) $$\mathrm{OIT}={\mathrm{OIT}}_{0\;}\cdot \;e^{-k^{\prime }D}$$

where *k*′ is the oxidation rate constant depending on the irradiation dose (*k*′= *k/D_r_*, where *D_r_* is the dose rate).

Equations (5) and (6) describe the aging process of ethylene-based materials in ionizing radiation fields using the measured OIT values and the kinetic model presented above.

Several cases of application of this simple kinetic model are discussed below.

#### 3.2.1. Evaluation of the Remaining Service Life (*t_x_*) When the Elapsed Aging/Service Time Is Known

As previously mentioned, the lifetime, *t_x_*, of a polymeric material can be assumed to be the time required for total consumption of the incorporated antioxidant, i.e., the total aging duration required to obtain a zero value for OIT under specific aging conditions.

Equation (5) can be used for *t_x_* evaluation if the *k* value is known. It can be calculated from Equation (5) if two OIT values are measured during two different aging periods, e.g., OIT_0_ for initial (unaged) material and OIT_*t*_ at aging time *t*.

(7) $$k=\frac{1}{t}\cdot \ln\frac{{\mathrm{OIT}}_{0}}{{\mathrm{OIT}}_{t}}$$

By imposing a limit condition for OIT_*t*_ (OIT_*t*_ →0) in Equation (7), meaning the end of life of the polymeric material, the service life (*t_x_*) can be calculated. However, using an exactly zero value makes Equation (7) mathematically impossible to solve, so a low OIT value must be imposed as a limit, e.g., 10 s (0.167 min).

(8) $$t_{x}=\frac{1}{k}\cdot \ln\frac{{\mathrm{OIT}}_{0}}{0.167}$$

Using different values, i.e., 100 s, will not result in significant differences in the evaluated lifetime. However, imposing very low OIT values, such as 10^−10^ s, results in considerably longer lifetime values, although it is reasonable to maintain near-practically measurable values for OIT limit. Consequently, the estimated lifetime will be slightly lower than the accepted value from mechanical tests (e.g., time to reach 50% elongation), enabling a safety reserve. A more extensive discussion on the correlation of the OIT values with the mechanical properties of polymeric materials will presented in a forthcoming paper.

#### 3.2.2. Evaluation of the Service Life (*t_x_*) of Polymeric Insulation When the OIT_0_ Value Is Not Known

An essential advantage of this simple kinetic model is that it enables the evaluation of the residual lifetime of material, even if the initial value of oxidation induction time (OIT_0_) is not available. In such a case (which is more frequent in practice), it is enough to measure two OIT values at two different moments during the service *t*_1_ and *t*_2_ (*t*_1_ can be measured, even at the moment of service start). Equations (9)–(11) are applicable in this case for the expression of the kinetic model, rate constant, and remaining lifetime calculations, respectively. Another advantage of the application of Equations (9)–(11) is that the OIT_0_ value would be not representative of materials stored for long periods before use, such as cables in storage.

(9) $${\mathrm{OIT}}_{t_{1}}={\mathrm{OIT}}_{t_{2}}\cdot \;e^{-k\left(t_{1}-t_{2}\right)}\;$$

(10) $$k=\frac{1}{t_{2}-t_{1}}\ln\frac{{\mathrm{OIT}}_{t_{1}}}{{\mathrm{OIT}}_{t_{2}}}$$

(11) $$t_{x}={\;t}_{1}+\frac{1}{k}\ln\frac{{\mathrm{OIT}}_{t_{1}}}{{\mathrm{OIT}}_{t_{x}}}$$

#### 3.2.3. Evaluation of the Maximum Supported Dose (*D_x_*) for a Cable in Use

For the practical cases wherein the dose is carefully monitored, such as nuclear power plants or particle accelerators, a similar first-order kinetic model can be used, whereby the time is replaced by the integral dose, *D*. The value *k*′ can be calculated (Equation (12)) if the OIT values at two different irradiation doses are known: for the cable in the initial state (*D*_0_) and the same material aged at dose *D*, respectively.

(12) $$k^{\prime }=\frac{1}{D}\cdot \ln\frac{{\mathrm{OIT}}_{0}}{{\mathrm{OIT}}_{D}}$$

By imposing a limit condition of OIT_*D*_ close to zero, the maximum supported dose can be calculated if the OIT_0_ and OIT_*D*_1__ or two OIT values at two different doses, *D*_1_ and *D*_2_, are known, using Equations (13) or (16), respectively.

(13) $$D_{x}=\frac{1}{k^{\prime }}\cdot \ln\frac{{\mathrm{OIT}}_{0}}{0.167}$$

Similar to the above, another approach to the simple kinetic model allows for the evaluation of the maximum supported dose without the need to know the OIT value for the cable in its initial state but only the OIT_*D*_1__ and OIT_*D*_2__ values obtained on two similar cable samples irradiated under the same conditions at two different doses (*D*_1_ and *D*_2_; *D*_2_ > *D*_1_).

(14) $${\mathrm{OIT}}_{D_{1}}={\mathrm{OIT}}_{D_{2}}\cdot \;\frac{e^{-k^{\prime }D_{1}}}{e^{-k^{\prime }D_{2}}}$$

(15) $$k^{\prime }=\frac{1}{D_{2}-D_{1}}\ln\frac{{\mathrm{OIT}}_{D_{1}}}{{\mathrm{OIT}}_{D_{2}}}$$

(16) $$D_{x}=D_{1}+\frac{1}{k^{\prime }}\ln\frac{{\mathrm{OIT}}_{D_{1}}}{{\mathrm{OIT}}_{D_{x}}}$$

### 3.3. Examples of Application of the Kinetic Model on Various Ethylene-Based Electrical Insulation Materials

#### 3.3.1. LDPE/XLPE Insulation Materials

**Figure 3:**
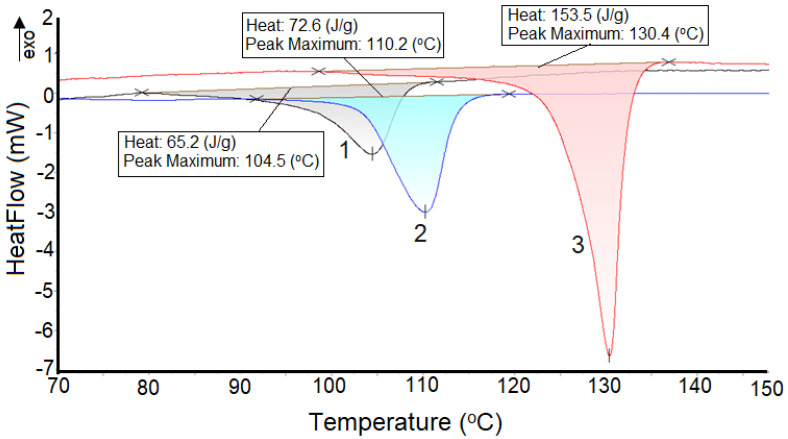
Melting endotherms from three different polyethylene materials: 1—XLPE insulation (from I&C cable); 2—LLDPE insulation (from multiconductor instrumentation); 3—HDPE insulation (from multiconductor instrumentation).

An example of the use of DSC as an analytic tool, based on crystallinity melting, for easy discrimination between different ethylene/substituted ethylene polymers is shown in Figure 3. The melting peaks are located at temperatures characteristic of the respective types of polymers. Note that FTIR spectra already confirmed the polyethylene nature of these polymers. Hence, DSC and FTIR spectroscopy can be used as a rapid quality test. The corroboration of the information inferred by both techniques enables accurate establishment of the polymer type, the fluctuation of properties from one batch to another, and an eventual substitution of the suitable polymer during the fabrication process.

**Figure 4:**
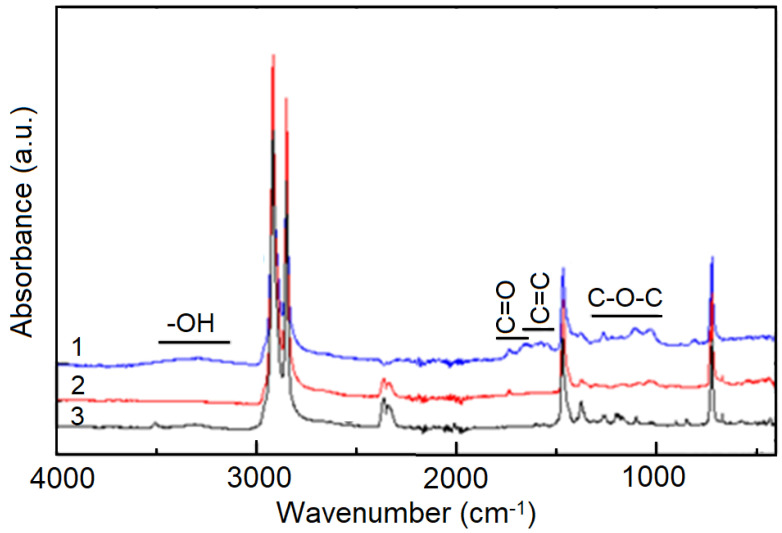
Typical ATR-FTIR spectra obtained on LDPE-based materials (HV cable): 1—Sheath, outer part (visually shows traces of photo-oxidation, i.e., color change); 2—sheath, inner part; 3—insulation.

Oxidized polyethylene (aged) has absorption bands in the spectral region of 3500–3300 cm^−1^ due to OH groups in hydroperoxides and alcohols, 1700–1780 cm^−1^ (carbonyl or carboxyl C=O groups), 1150–1050 cm^−1^ (C−O−C from oxidation products), 1640–990, 910, and 890 cm^−1^ (double bonds of different types) (Figure 4).

Table 1 shows an example of a kinetic model application in the case of the same material exposed to irradiation (γ^137^Cs) in different sample forms, namely (i) thin strap (thin strap previously sampled from cable insulation by a turning machine), (ii) bulk-free (sampled from an irradiated cable fragment with free ends), and (iii) bulk-encapsulated (sampled from an irradiated cable fragment with wax-capped ends). The rate constant (*k*′) is lower for the sample irradiated as a strap at a lower dose rate as compared to bulk samples due to the free access of oxygen over the whole strap surface and the dose-rate effect in the presence of oxygen [48]. There is also a certain difference between the degradation rates of the samples irradiated as bulk with or without limited contact with air; the prevention of air access resulted in a lower degradation rate and, consequently, in a longer lifetime. The latter case is probably closer to the inner parts (far from the ends) of a real cable, whereas the former (bulk air or uncapped) would be close to the situation of real cable ends (at the connectors). As a result, except for the surface layer, which is in direct contact with the air, irradiation occurs mainly in the absence of oxygen. Hence, crosslinking is dominant. After irradiation under these conditions, a relatively large amount of free radicals may remain trapped in the polymer, leading to slow post-irradiation oxidation as they combine with the oxygen diffused slowly into the bulk [10,48]. The presence of trapped species in irradiated polymers can be easily evidenced in chemiluminescence by the initial signal increase assigned to the sample’s history [35].

The effects of the dose rate are illustrated in the case of polymeric materials from witness cables aged under real service conditions (Table 2) in the SPS CERN accelerator.

This behavior can be understood in terms of antioxidant consumption through two competing processes. One is the reaction with free peroxy radicals, for which the rate depends on available oxygen (diffusion-limited), and the other is antioxidant consumption through direct reaction with the radiation-induced alkyl free radicals of the polymer. Increased dose rates can enhance the reaction share in the latter process, resulting in accelerated antioxidant consumption, lower OIT values, and lower lifetime values.

Table 2 also shows that the different insulation materials result in significantly different OIT_0_ values; the highest OIT_0_ is observed for black insulation, whereas lower values are observed for green/yellow insulation. These different values could be due to different levels of incorporated antioxidants, as well as possible influences of the dye additives on thermal oxidation kinetics [2]. Furthermore, the specific influence of the additive systems can also explain the slight differences observed in the radiation behavior of the four insulation materials.

**Figure 5:**
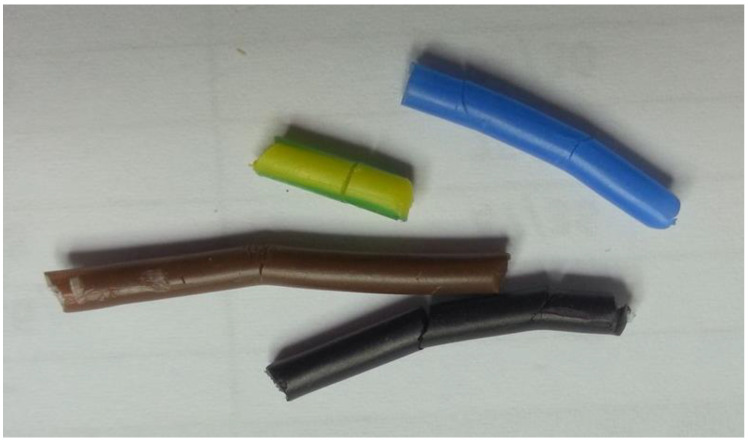
Appearance of cracks in XLPE insulations (IC1 cable) after three years of irradiation in SPS.

The zero OIT values measured for the samples irradiated at 105 kGy and 144 kGy (Table 2) are in agreement with the observation that these samples are seriously damaged by irradiation, presenting cracks (Figure 5) and friability. This confirms the viability of the proposed kinetic model.

**Figure 6:**
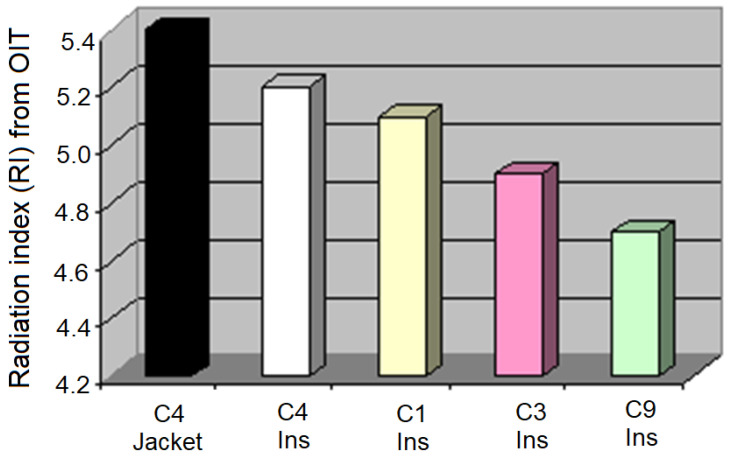
RI values of irradiated LDPE cable materials (strap, γ^137^Cs, D_r_ = 0.4 kGy/h); C9 was irradiated as bulk (free ends).

A frequently used parameter for characterization of the radiation strength of a material is the *radiation index* (RI), which is defined in IEC 60544-4 [49] as the log_10_ of the absorbed dose in gray (rounded to two significant digits) at which the elongation at break is reduced to 50% of its initial value under specified conditions of irradiation and tests. As we assumed that the mechanical properties of a material should fail after the OIT reaches a zero value, it is reasonable to assume that RI can be evaluated in this manner from OIT measurements. Hence, considering the *D_x_* values calculated from OIT for several polyethylene insulation materials subjected to gamma irradiation under controlled conditions (Figure 6), the RI values calculated with the formula RI = log_10_*D_x_* are similar to those previously reported for PE materials by Tavlet and Schönbacher based on mechanical tests [28,29] using similar dose and dose-rate conditions. The practical importance of these results is that using thermal analysis methods instead of mechanical tests for degradation diagnosis and lifetime prognosis makes it possible to reduce both the amounts of required samples and laboratory time and to increase the amount of information inferred by the measurements. Hence, improved condition monitoring of in-use cables can be achieved.

#### 3.3.2. EPR Copolymer Insulation Materials

Ethylene-propylene copolymers are a class of synthetic rubbers obtained by copolymerizing ethylene and polypropylene, possibly in combination with other chemical compounds (diene, in a proportion of a few percent) to form ethylene-propylene-diene monomer rubber (EPDM). Both EPR and EPDM polymers are of interest for the production of cable insulation due to their low production costs and their interesting properties, such as mechanical flexibility, thermal stability, good dielectric properties in humid conditions, high resistance to tree formation and corona effect, low hydrophobicity index, abrasion resistance, and high thermal range of use (−60 °C and + 160 °) [5,6,19,50,51,52]. Due to its rubber-like properties, EPR is used in the manufacture of many distribution cables up to 35 kV [53] or even 150 kV in direct current (DC) applications [54], cables for nuclear power plants [55,56], etc.

**Figure 7:**
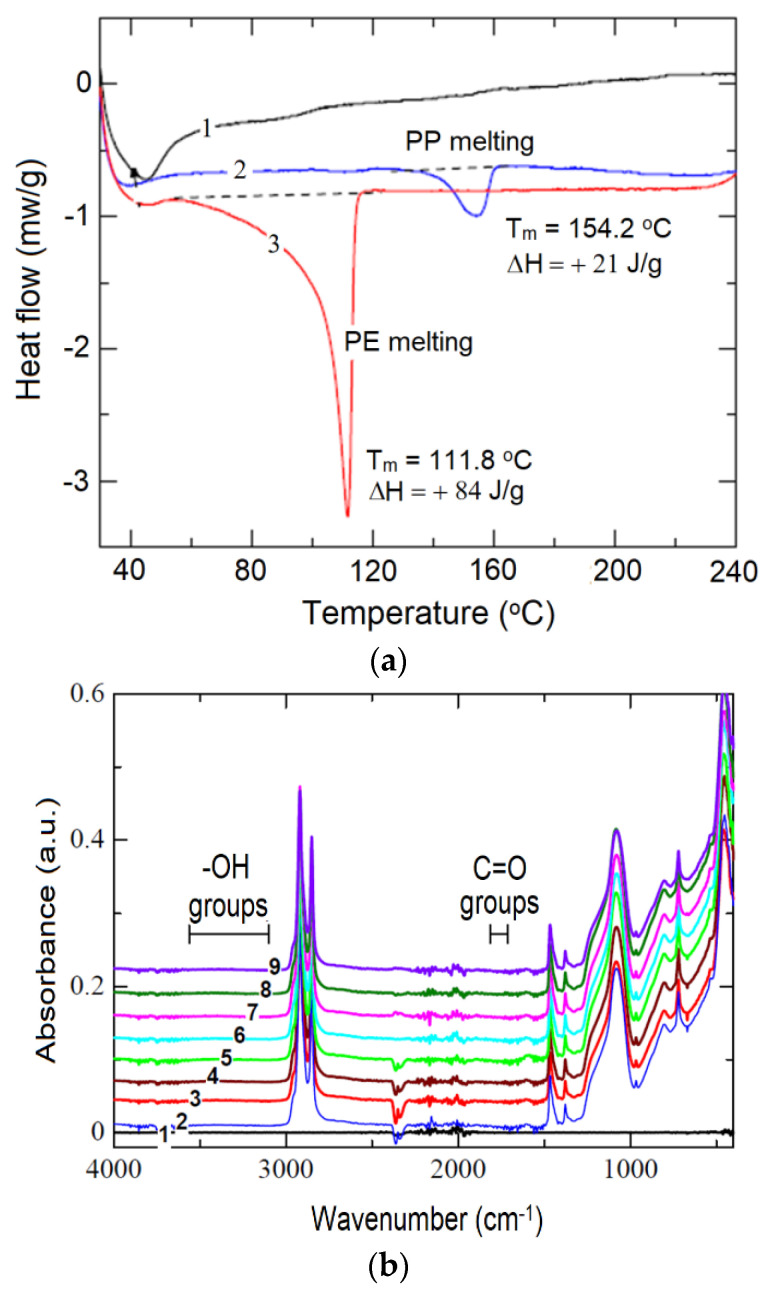
(**a**) Comparative non-isothermal DSC curves (β = 5 K/min., air): 1—EPR insulation; 2—EPR sheath; 3—LDPE insulation. (**b**) ATR/FTIR spectra of irradiated EPR insulation at different doses under different conditions: (1)—reference (ATR crystal); (2)—0 kGy; (3)—9.6 kGy (bulk-free ends); (4)—28.8 kGy (bulk-free ends); (5)—96 kGy (bulk-free ends); (6)—310 kGy (bulk-free ends); (7)—310 kGy (bulk-wax-encapsulated ends); (8)—630 kGy (bulk-free ends); (9)—630 kGy (bulk-wax-encapsulated ends).

The cables used at CERN, known as “*EPR cables*”, contain EPR in both the insulation and the sheath, with differing chemical natures. The sheath has a high PP content and a higher level of crystallinity. The PP/ethylene ratio is high in semicrystalline sheath materials. ATR-FTIR and non-isothermal DSC methods allow for differentiation between these two types of EPR-based cable components. DSC non-isothermal measurements show a softening peak at 153 °C (Figure 7a), and ATR/FTIR spectroscopy shows a high absorption band at 1380 cm^−1^ (due to bending vibration of the C-H bonds in CH_3_ (Figure 7b).

As in the case of LDPE, the OIT values of EPR-based insulation decrease exponentially with the irradiation dose (Table 3), suggesting the possibility of applying the proposed kinetic model to evaluate the lifetime of these materials. For the validation of the model, samples of HV cable with EPR insulation were subjected to irradiation at different irradiation doses and dose rates with both free and wax-encapsulated ends.

The methodology for assessing the maximum dose and lifetime for EPR materials is similar to that applied to LDPE-based materials. A similar cable with EPR insulation, with free access to oxygen (free ends), was irradiated under use conditions in SPS for one year. As the total dose received by the cable during this period was unknown, the life of this cable could be estimated based on OIT values under specific use conditions.

#### 3.3.3. EVA Copolymer Insulation Materials

Due to their elastic properties, poly(ethylene-co-vinyl) acetate (EVA) copolymers, which are widely used in the production of wires and cables, incorporate a high concentration of stabilizers and mineral fillers for both high stability and fire resistance without halogen [57].

**Figure 8:**
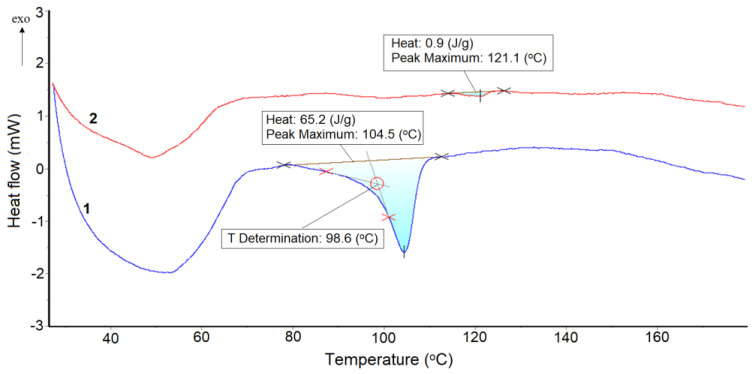
DSC curves of XLPE insulation of IC1 cable (1) and EVA sheath (2) of IC2 cable in a nitrogen atmosphere (50 mL/min).

Figure 8 shows a comparison of the DSC curves for XLPE and EVA materials. Such curves enable easy discrimination between crystalline polymers, such as PE, and amorphous copolymers, such as EVA in our case. The melting endotherm is weak in the case of the copolymer (with Tm at ~93 °C) because of its typically low crystallinity. The vinyl-acetate (VA) content was graphically estimated at 16% based on Tm values using the relationship proposed by Son and Choi (2019) [58].

**Figure 9:**
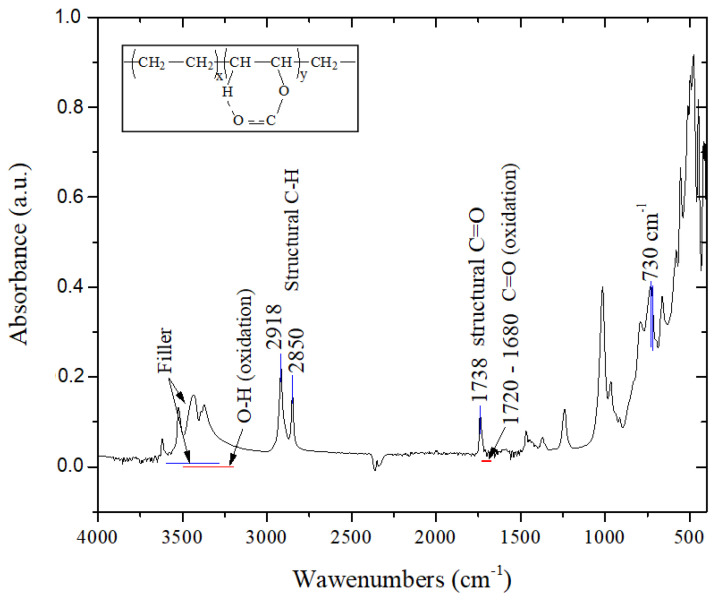
ATR-FTIR spectrum of the EVA jacket of IC2 cable with significant bands. Inset: internal hydrogen bond in vinyl-acetate groups.

ATR-FTIR spectroscopy (Figure 9) confirms the DSC data. The most specific band of EVA copolymer is at 1738 cm^−1^, assigned to the carbonyl group in esters. This structural band makes the ATR/FTIR method challenging to apply when monitoring the degradation of these materials.

Oxidation induced by stress factors results in weaker peaks located at 1680–1720 cm^−1^. The group of four peaks located between 3300 and 3600 cm^−1^ and the intense band between 500 and 700 cm^−1^ are due to the mineral filler. As the vinyl acetate comonomer is free of OH groups, the spectrum of the copolymer should be free of absorption bands in the region of 3200–3500 cm^−1^; however a small, straight band is observed at around 3430 cm^−1^ [59], especially for copolymers with high VA content. These peaks are the result of intramolecular hydrogen bonds within the VA moiety (see the inset in Figure 9 for hydrogen bonds). However, even if such a peak exists, it is probably masked by more intense bands of the mineral filler. Furthermore, based on the IR spectra, the VA content of 17% was evaluated from the ratio of the peak areas at 730 cm^−1^ and 720 cm^−1^ [59], which is quite close to the value determined from DSC measurements.

**Figure 10:**
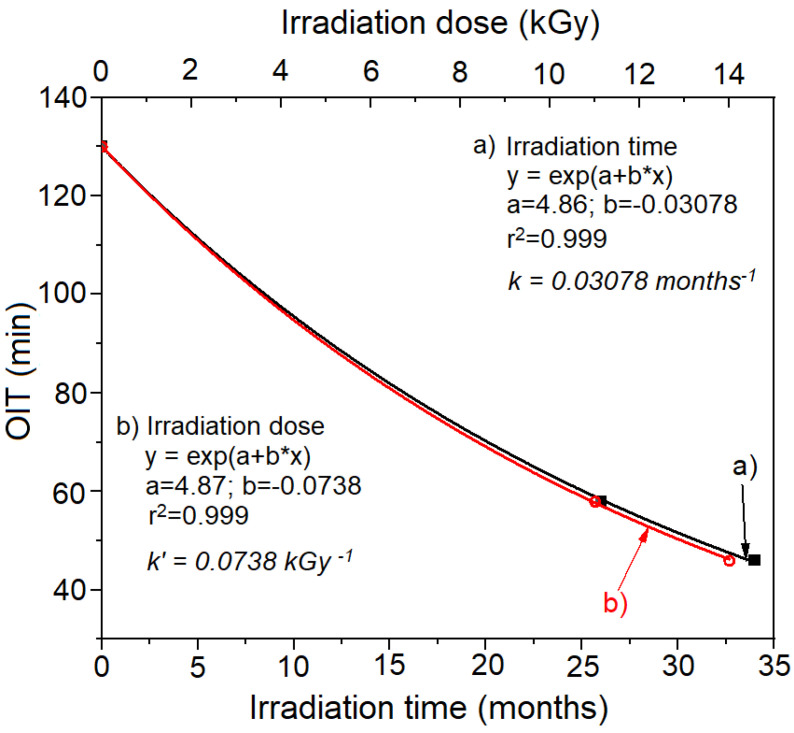
Exponential OIT decrease during irradiation in the CERN—SPS accelerator. (**a**) OIT vs. irradiation time; (**b**) OIT vs. irradiation dose.

Similar to the polymeric materials presented above (i.e., LDPE, XLPE, and EPR), EVA exhibits the same behavior in terms of OIT values decreasing over time (Figure 10a), irrespective of irradiation dose (Figure 10b). This behavior suggests that the proposed kinetic model can be applied to these materials for evaluation of lifetime and maximum supported irradiation dose (Table 4).

### 3.4. Radiation-Induced Oxidation Profiles in Cable Depth by OIT Measurements

**Figure 11:**
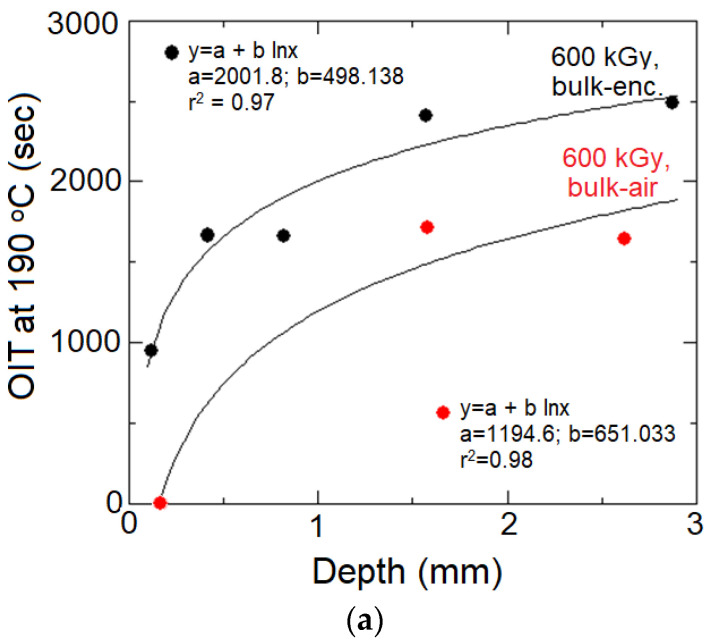

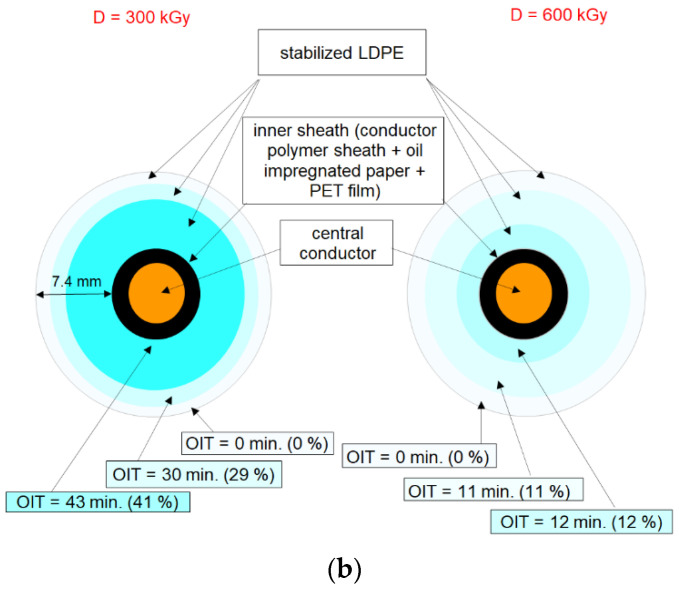
(**a**) Longitudinal oxidation profiles from OIT measurements in PE insulator of C3 cable (isothermal DSC mode, 190 °C, air). (**b**) Transversal oxidation profiles from OIT measurements in PE insulator of C4 cable; the percentages in parentheses indicate the value of the ratio $\frac{{\mathrm{OIT}}_{D}}{{\mathrm{OIT}}_{0}}\times$100 (isothermal DSC mode, T = 190 °C).

In the case of irradiated thick insulation samples, the radiation-induced oxidation is typically heterogeneous due to the influence of atmospheric oxygen. Figure 11a shows that OIT data vs. sampling depth fit logarithm functions. Furthermore, the OIT values from the wax-encapsulated samples (C3 cable bulk-encapsulated) are systematically higher than those of the non-encapsulated sample. Similar data from another HV cable (C4 cable) are presented in Figure 11b.

The effect of the irradiation dose in general diminution of OIT values is clearly observed. Representative OIT values illustrate the heterogeneous degradation of the insulation material in sample depth. The 5 mm sheath in oil-impregnated paper and a PET layer adherent to the PE insulator explain the reduced oxidation in the inner region compared to the outer region, where the OIT value was zero.

**Figure 12:**
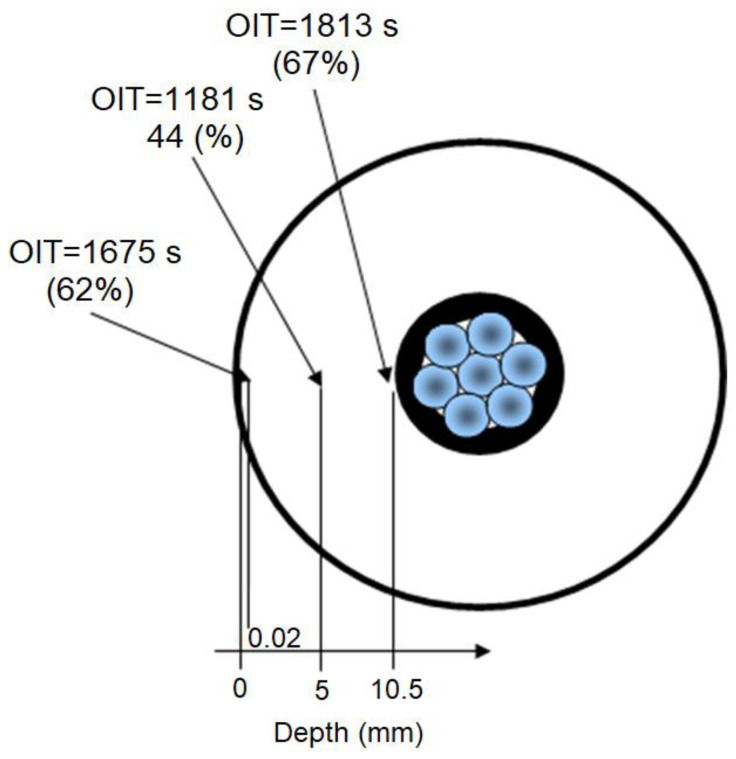
Transversal oxidation profile from OIT measurements of the C1 cable. Irradiation dose: 600 kGy (bulk-air). The percentages in parentheses indicate the value of the ratio $\frac{{\mathrm{OIT}}_{D}}{{\mathrm{OIT}}_{0}}\times$100 (isothermal DSC mode, 190 °C, air).

The tightly packed structure of C1 cable appears to provide good protection against the ingress of air around the insulator, with the black sheaths covering both sides of the insulator, probably as oxygen barriers. As a result, the OIT values found for both encapsulated and non-encapsulated cables are similar, with significant OIT values measured on both sides of the insulator (Figure 12). This behavior is different from that observed for cables C3 and C4, which presented zero values of OIT on the outer side of the insulator, even at a relatively long distance (2–3 cm) from the cable end.

**Figure 13:**
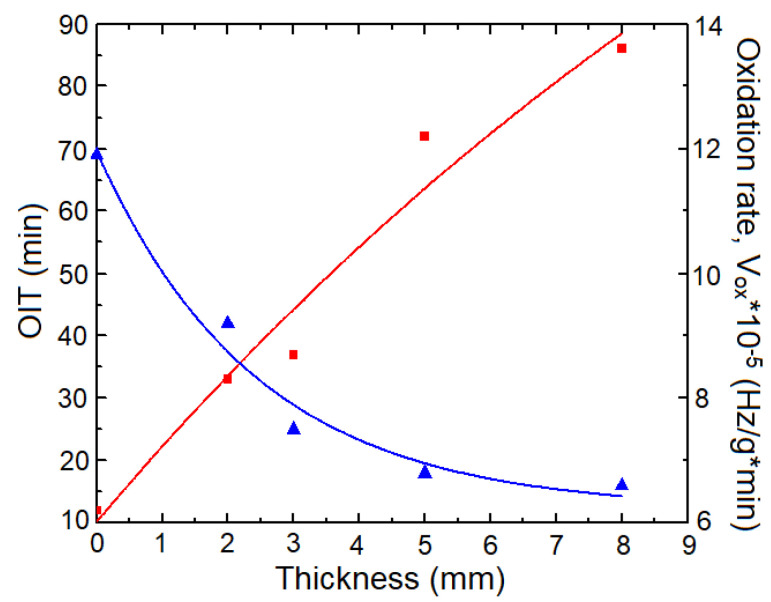
Oxidation induction time and oxidation rate vs. depth in PE-insulating material (isothermal CL, 200 °C, air).

CL measurements (Figure 13) lead to similar conclusions, showing that the oxidation degree decreases from the surface in contact with air towards the inner material zones, following the direction of oxygen diffusion in the insulating material.

The results prove the capability of the DSC method to explore the oxidation profile of a bulk sample, evidencing the inhomogeneous oxidation in cable depth. In the case of cable insulation, the sheaths, the gas-tight connectors, and the jackets contribute to inhomogeneous oxidation by hindering air access. Considering the possible presence of inhomogeneous oxidation, the correct selection of the relevant samples from an aged cable is mandatory. For large-diameter insulators, such as in HV cables, it is necessary to analyze samples from both the external and internal surfaces of the insulator, as well as from the middle zone of the insulator in order to obtain the transversal oxidation profile following the electric field direction, which is most important for the insulator aging state.

### 3.5. DSC-CL Correlation

Chemiluminescence is a technique characterized by very high sensitivity in detection of the oxidation processes (CL: luminescence produced due to a chemical reaction). The emission of chemiluminescence is closely related to oxidation, a sufficiently exothermic process to produce excited molecular species with enough energy for deactivation, leading to light emission in the visible range [60]. The sampling is similar to DSC method, but the sample’s surface is explored with a photomultiplier tube coupled with electronic signal amplification systems, making these systems exceptionally sensitive [61].

Eliminating the contribution of other endothermic (molecular decomposition) or exothermic (crystallization) processes considerably simplifies the CL curve and contributes to increased accuracy of measurements [61]. However, the most important limitation of the method is the self-absorption of the CL signal in dark and/or high-mineral-charge materials.

**Figure 14:**
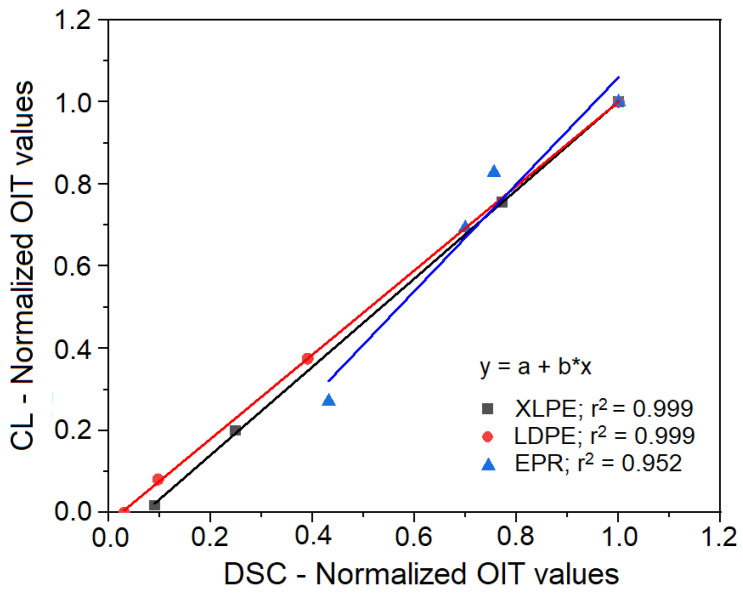
DSC—CL OIT correlation.

The experimental results obtained by DSC-CL comparative analysis performed on insulating cable materials (based on LDPE, XLPE, EPR, and EVA), showed an excellent correlation (r^2^ > 0.99) of data, especially on LDPE and XLPE materials (Figure 14). In the case of EPR and EVA samples, lower linear correlation (r^2^: 0.92–0.94) between the two types of measurements was observed. This can be explained either by the fact that the samples are not melted and the thermal contact with the heated element is poor or because the samples are opaque and, therefore, the amount of signal that reaches the CL photomultiplier tube is less than that for PE. Both techniques are suitable for aging characterization in an ionizing radiation environment for the polymeric materials used to produce the electric cable.

The CL method can provide clearer and richer information related to both the oxidation mechanism and the effect of stabilizers [35]. However, in the case of cable materials, its effectiveness is limited by their opacity or color. Thus, for current practical applications, the use of the DSC technique appears most beneficial because it also offers the opportunity to highlight feature transitions (e.g., melting temperature, glass transition, etc.), which can be used to identify the nature of the polymeric material.

## 4. Conclusions

We propose a kinetic model based on the exponential decrease in oxidation induction time (OIT) to evaluate the lifetime of electrical insulating polymeric materials in electrical cables in ionizing radiation environments. The model also allows for determination of the maximum radiation dose that the polymeric material can support.

This model has been tested on dozens of cable types with polymeric materials of different chemical nature (LDPE, HDPE, XLPE, PE foam, EPR, and EVA) in use in CERN’s particle acceleration systems. However, it can be extended to evaluate the lifetime of polymeric materials exposed to other stress factors, such as temperature, UV, natural aging, etc.

The results of the evaluation of the lifetime of polymeric materials from cables exposed to radiation environments using the proposed kinetic model correlate very well (>95%) with lifetime evaluation data resulting from mechanical testing (50% reduction in elongation at break). These results will be the subject of a future paper.

## Figures and Tables

**Table 1:** Degradation model parameters and lifetime evaluation of LDPE insulation of C1 cable (isothermal DSC measurements, T = 190 °C, air).

Irradiation Conditions	Dose (kGy)	Dose Rate (kGy/h)	OIT (min)	*k*′ (kGy^−1^)	*D_x_* (kGy)	*t_x_* (h)
Strap	0	0.4	45	4.27 × 10^−2^	131	328
	9.6		34			
	28.8		8.7			
	96		0.8			
Bulk-free ends	0	1.5	45	1.20 × 10^−3^	4629	3086
	310		23.5			
	630		25			
Bulk-wax-encapsulated	0	1.5	45	1.07 × 10^−3^	5196	3464
	310		29			
	630		26			

**Table 2:** Degradation model parameters and lifetime evaluation of XLPE insulation of IC1 cable (different colored multi conductors; Figure S5) irradiated in the CERN-SPS accelerator (isothermal DSC measurements, T = 200 °C, air).

Irradiation Time (Months)	Dose * (kGy)	OIT (min)				*t_x_* (Months)/*D_x_* (kGy)			
		XLPE Color							
		Black	Brown	Blue	Green-Yellow	Black	Brown	Blue	Green-Yellow
0	0	177	131	122	93	−	−	−	−
26	6	3.7	3.9	3.9	2.0	45/11	47/11	47/11	45/11
	11	35	41	33	3.6	107/47	140/60	120/53	48/21
	22	24	47	68	7.5	86/75	160/140	250/220	62/55
	26	8.3	5.3	7.4	5.7	56/55	50/55	57/60	56/60
	105	0	0	0	0	<26/<105			
34	11	3.5	3.7	2.9	2.2	53/15	59/17	57/16	55/16
	14	29	29	30	11	126/50	150/62	145/59	105/41
	24	65	39	55	5.6	250/167	184/136	267/198	74/55
	31	4.7	4.0	3.8	3.5	65/59	62/57	64/59	64/59
	144	0	0	0	0	<26/<105			

* Different irradiation positions in the CERN-SPS accelerator.

**Table 3:** Degradation model parameters and lifetime evaluation of EPR insulation of C2 cable (isothermal DSC measurements, T = 200 ° C, air; Figure S6).

Irradiation Conditions	Dose (kGy)	Dose Rate (kGy/h)	OIT (min)	*k*′ (kGy^−1^)	*D_x_* (kGy)	*t_x_*
Bulk-free ends	0	0.4	372	1.36 × 10^−2^	567	1418 h
	9.6		308			
	28.8		258			
	96		101			
Bulk-free ends	0	1.5	342	2.36 × 10^−3^	3266	8165 h
	310		143			
	630		94			
Bulk-wax-encapsulated	0	1.5	342	02.19 × 10^−3^	3520	8800 h
	310		238			
	630		90			
One year in SPS (bulk-free ends)	0	N/A	246	−	−	~22 years
	N/A		177			

**Table 4:** Degradation model parameters and lifetime evaluation of EVA sheath of a multiconductor IC2 cable irradiated in the CERN-SPS accelerator (isothermal DSC measurements, T = 220 ° C, air; Figure S7).

Irradiation Time (Months)	Dose * (kGy)	OIT (min)	*t_x_* (Months)/*D_x_* (kGy)
0	0	130	−
26	6	8	61/14
	11	58	206/90
	22	11	75/58
	26	25	100/100
	105	33	121/246
34	11	7	69/21
	14	46	214/90
	24	8	76/57
	31	19	113/106
	144	1	58/246

* Different irradiation positions in the CERN-SPS accelerator.

## Data Availability

The data presented in this study are available on request from the corresponding author.

## Supplementary Materials

The following supporting information can be downloaded at: https://www.mdpi.com/article/10.3390/polym14122357/s1, Figure S1: Structure of C1 cable (General purpose, High Voltage – HV- coaxial cable); Figure S2: Structure of C4 cable (General purpose, HV/HF coaxial cable); Figure S3: Structure of C3 cable (General purpose, HV/HF—High Frequencies—coaxial cable); Figure S4: Structure of C9 cable (General purpose, I&C—Instrumentation and Control- cable); Figure S5: Structure of IC1 cable (General purpose, I&C—Instrumentation and Control- cable); Figure S6: Structure of C2 cable (General purpose, HV, coaxial cable); Figure S7: Structure of IC2 cable (General purpose, I&C—Instrumentation and Control-cable).
