# Supplementary material for: On the Use of Oxidation Induction Time as a Kinetic Parameter for Condition Monitoring and Lifetime Evaluation under Ionizing Radiation Environments
Source: Polymers (Basel). 2022 Jun 10;14(12):2357. doi: 10.3390/polym14122357 (PMC9229975; doi:10.3390/polym14122357)
Supplement: Supplementary file 1 [file polymers-14-02357-s001.zip › polymers-1737075-supplementary.pdf]

Supplementary material

On the use of oxidation induction time as a kinetic parameter for condition monitoring and lifetime evaluation under ionizing radiation environments

Eduard-Marius Lungulescu[1,*], Radu Setnescu[1,2], Sorin Ilie[3], Mauro Taborelli[4],

[1]National Institute for Research and Development in Electrical Engineering ICPE-CA, 313, Splaiul Unirii, 030138 Bucharest, Romania; radu.setnescu@icpe-ca.ro (R.S.)

[2]Department of Advanced Technologies, Faculty of Sciences and Arts, Valahia University of Târgoviște, 13 Aleea Sinaia, 130004 Targoviste, Romania

[3] Former head of TS-MME/CCS in CERN; sorins712@gmail.com (S.I.)

[4] European Organization for Nuclear Research (CERN), 1211 Geneva 23, Switzerland; mauro.taborelli@cern.ch (M.T.)

*Correspondence: marius.lungulescu@icpe-ca.ro (E.-M.L)

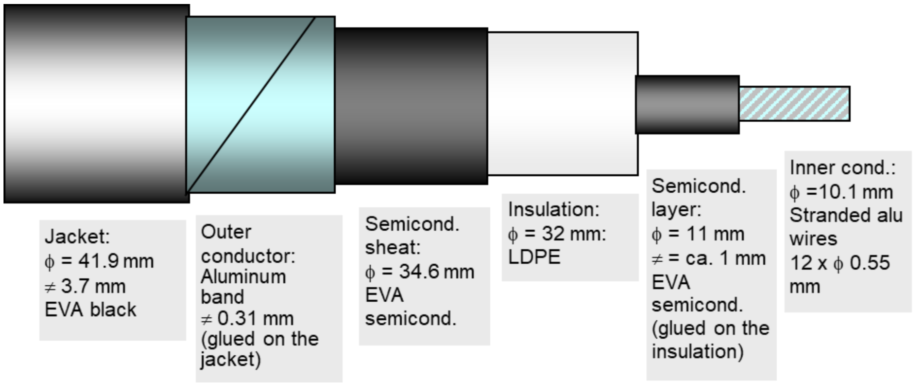

Figure S1. Structure of C1 cable [General purpose, High Voltage - HV- coaxial cable]

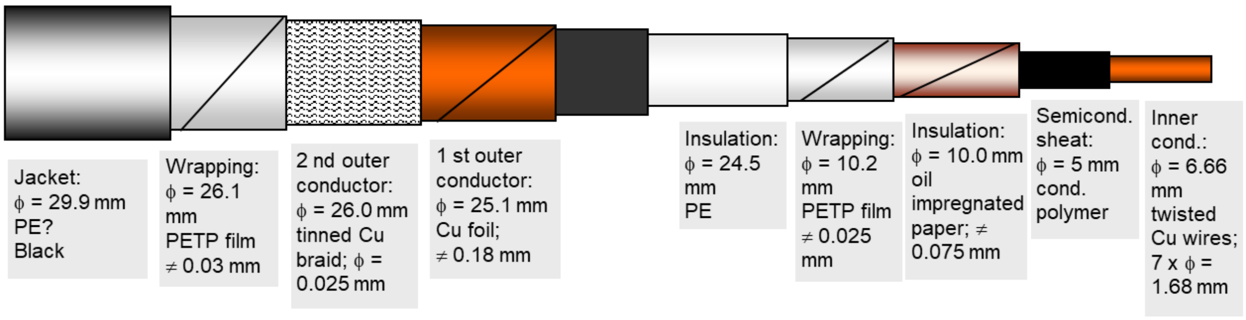

Figure S2. Structure of C4 cable (General purpose, HV/HF coaxial cable)

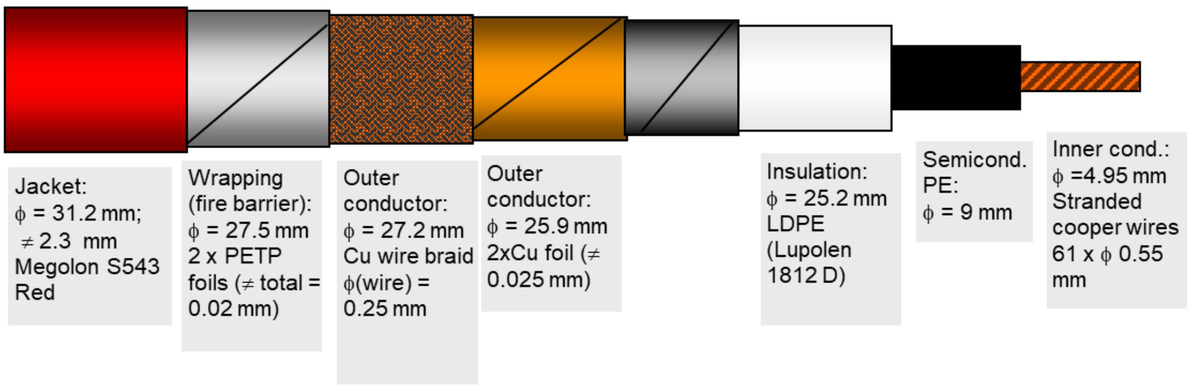

Figure S3. Structure of C3 cable (General purpose, HV/HF - High Frequencies - coaxial cable)

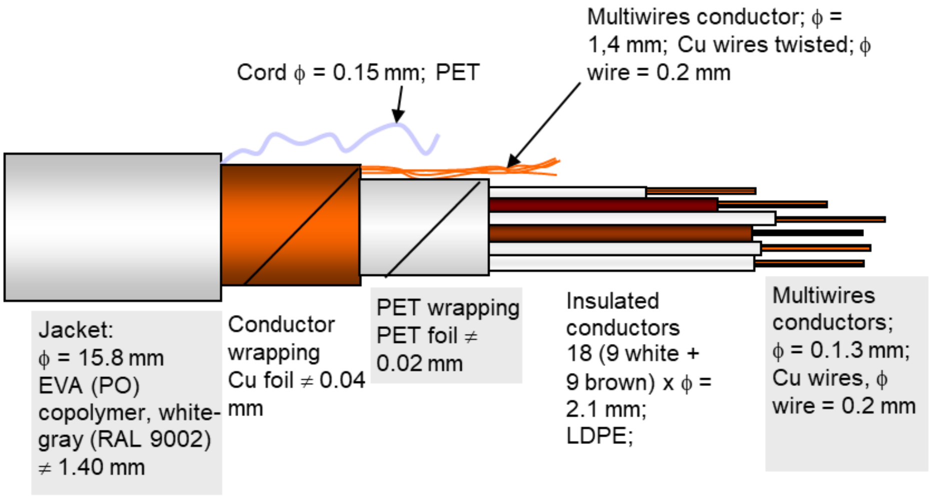

Figure S4. Structure of C9 cable (General purpose, I&C - Instrumentation and Control- cable)

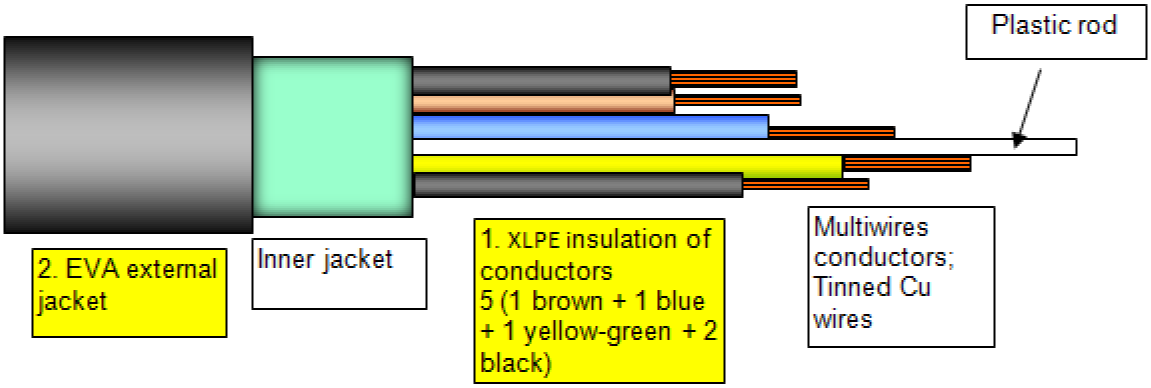

Figure S5. Structure of IC1 cable (General purpose, I&C - Instrumentation and Control- cable)

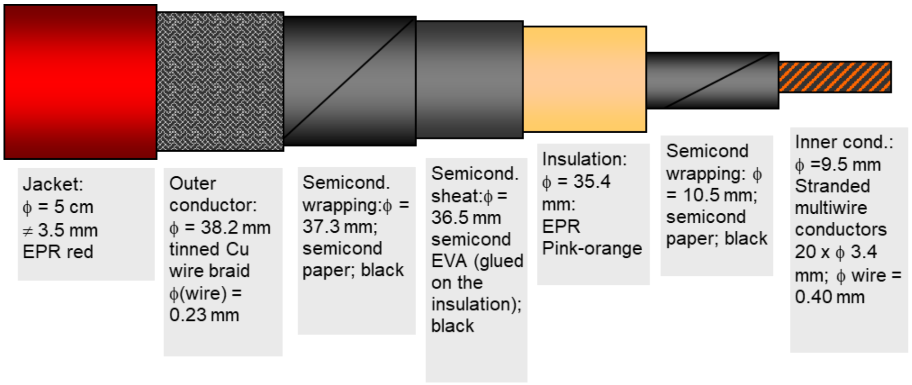

Figure S6. Structure of C2 cable (General purpose, HV, coaxial cable)

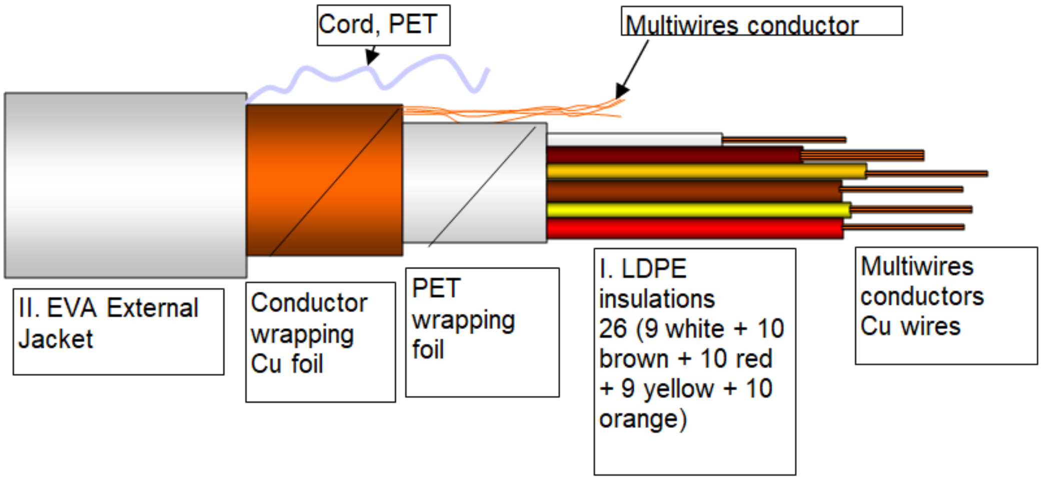

Figure S7. Structure of IC2 cable (General purpose, I&C - Instrumentation and Control- cable)
